# Dynamic genetic diversity and population structure of *Coreiusguichenoti*

**DOI:** 10.3897/zookeys.1055.70117

**Published:** 2021-08-11

**Authors:** Dongqi Liu, Feng Lan, Sicai Xie, Yi Diao, Yi Zheng, Junhui Gong

**Affiliations:** 1 Sichuan Province Key Laboratory of Characteristic Biological Resources of Dry and Hot River Valley, Panzhihua University, Panzhihua, 617000, China Panzhihua University Panzhihua China; 2 Upper Changjiang River Burean of Hydrological and Water Resources Survey, Chongqing, 400000, China Upper Changjiang River Burean of Hydrological and Water Resources Survey Chongqing China

**Keywords:** *
Coreius guichenoti
*, genetic diversity, mtDNA, population structure

## Abstract

To investigate the genetic effects on the population of *Coreiusguichenoti* of dam constructions in the upper reaches of the Yangtze River, we analyzed the genetic diversity and population structure of 12 populations collected in 2009 and 2019 using mitochondrial DNA (mtDNA) control regions. There was no significant difference in genetic diversity between 2009 and 2019 (*P* > 0.05), but the population structure tended to become stronger. Genetic differentiation (FST) among five populations (LX, BB, YB, SF and JA) collected in 2009 was not significant (*P* > 0.05). However, some populations collected in 2019 were significantly differentiated (*P* < 0.05), indicating that the population structure has undergone change. A correlation analysis showed that the genetic diversity of the seven populations collected in 2019 was significantly negatively correlated with geographical height (*r* = −0.808, *P* = 0.028), indicating that the populations at high elevations were more vulnerable than those at low elevations. In order to prevent the further decrease of genetic diversity and population resources, some conservation and restoration suggestions, such as fish passage and artificial breeding, are put forward.

## Introduction

*Coreiusguichenoti* (Sauvage & Dabry de Thiersant, 1874) (Cyprinidae, Cypriniformes) is one of the endemic fishes and an important economic fish in the upper reaches of Yangtze River in China (Ding 1994; Chen et al. 2002). Due to dam construction and environmental degradation, including the destruction of spawning and feeding grounds, the population of *C.guichenoti* has declined significantly (Cheng et al. 2015). In the evaluation of the conservation status of wild vertebrates in China, *C.guichenoti* is at the extremely endangered level (Jiang et al. 2016). Liu (2004) quantitatively analyzed the priority conservation order of endemic fish in the upper reaches of the Yangtze River by using the genetic loss coefficient and species value coefficient, and the results showed that *C.guichenoti* has reached the third-class conservation level.

**Figure 1.**
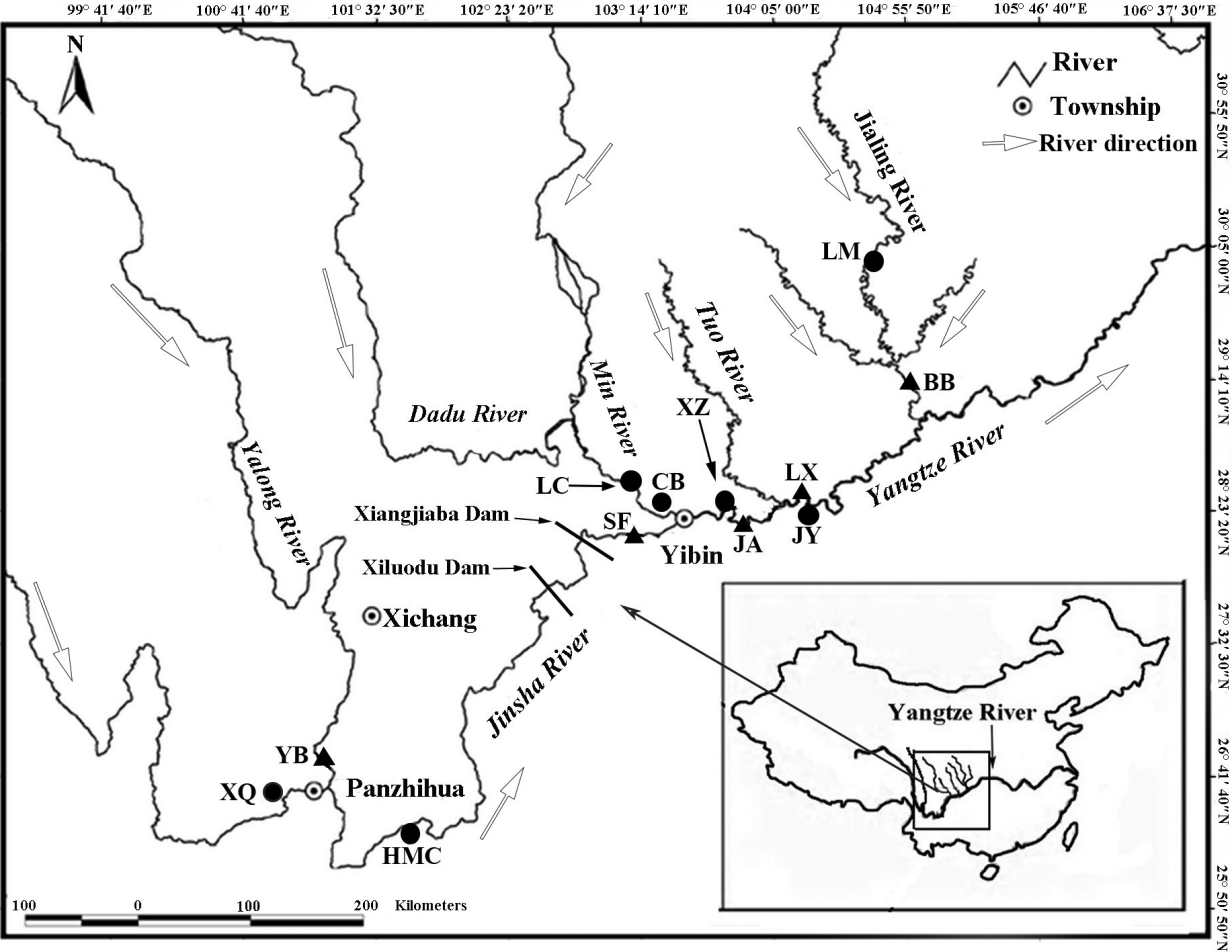
Sampling localities of China (solid triangles indicate sites in 2009; solid circle indicate sites in 2019) of *C.guichenoti*. For full names of populations, see Table 1.

*Coreiusguichenoti* were often found in fast currents of rivers and streams near gravel and rock crack habitats, and they feed on small fish (Chen et al. 2002). When rivers flood, the fish usually swim upstream and spawn eggs, and the larvae develop from March to May (Ding 1994). Therefore, a relatively long, continuous river is necessary for embryonic development and larval growth of *C.guichenoti*. However, this requirement on the natural history of *C.guichenoti* is in contradiction with the construction of cascade hydropower stations in the middle and upper reaches of the Yangtze River (Liu et al. 2020). In the past 20 years, a series of hydropower projects have been built in the upper reaches of the Yangtze River (Fig. 1). In 2007 and 2009, Xiluodu Dam and Xiangjiaba Dam were built in the lower reaches of Jinsha River, and these hydropower development projects have no facilities for fish to pass through. The survival of wild fish is under serious threat. In particular, dam construction will not only affect the spawning and development environment of the fish, but also hinder their up-and downstream migration (Liu et al. 2018). The habitat of species will become fragmented, and the genetic links between the population and communication will be interrupted, which may lead to genetic bottlenecks and inbreeding. Therefore, the genetic diversity of wild populations may be reduced. Wild species must have an available pool of genetic diversity if they are to survive the environmental changes that exceed the limits of developmental plasticity. If this is not the case, species extinction seems inevitable (Liu et al. 2015). For endangered species, it is essential to maintain as much genetic variation as possible to increase the chances of their populations recovering (Hedrick et al. 2000). Therefore, effective conservation measures should be developed to protect natural populations of *C.guichenoti*.

**Table 1.** Population geographic location, sample number and time for each site sampled.

Code	Locality	N_1_	N_2_	Elevation (m)	Sample time
LX	Luxian	24	21	245	Jun 2009
BB	Beibei	21	19	212	Sep 2009
YB	Yanbian	22	20	1003	Aug 2009
SF	Shuifu	35	28	431	May 2009
JA	Jiangan	33	28	293	Jun 2009
XZ	Xuzhou	45	29	284	Jun 2019
JY	Jiangyang	21	20	247	Aug 2019
LC	Longchi	36	26	323	Sep 2019
LM	Liemian	30	30	294	Oct 2019
CB	Caiba	45	35	285	May 2019
XQ	Xiqu	26	22	1115	Nov 2019
HMC	Hongmenchang	39	30	902	May 2019
Total		377	308		

N_1_: sample number before selected; N_2_: sample number after selected.

In view of the current and potential anthropogenic negative factors affecting the population of *C.guichenoti*, it is necessary to carry out long-term monitoring of the genetic status of wild population dynamics. Especially, after the construction of Xiluodu and Xiangjiaba dams, what is the genetic diversity of the wild population of *C.guichenoti*? Have these dams affected the population structure of *C.guichenoti*, and to what extent?

Previous studies have tried to evaluate genetic diversity and population structure of *C.guichenoti* from other sections of the Yangtze River (Cheng et al. 2015; Zhang and Tan 2010), but the results of these studies are inconsonant. Cheng et al. (2015) indicated low levels of diversity and strong unidirectional gene-flow between the genetic populations, but high-genetic diversity was reported by Zhang and Tan (2010). Additionally, a comparative study of the *C.guichenoti* in a large number of samples from other periods (especially after two dam constructions since 2009) has not been reported. In this study, we used mitochondrial DNA (mtDNA) control region to analyze the genetic diversity and structure of 377 individuals from 12 populations of *C.guichenoti*, which were collected from the upper reaches of the Yangtze River in 2009 and 2019. The population diversity and structure were compared between 2009 and 2019. Different groups were compared within the same sampling time, and among different populations in the same group. Understanding the genetic diversity and structure of historical and current populations will help to make the right management decisions and identify feasible conservation measures that contribute to the conservation and restoration of wild resources (Liu et al. 2020). Meanwhile, this study also provides important information for evaluating gene flow and genetic diversity of other species with a similar life history.

## Materials and methods

### Sample collection and DNA amplification

During the period of 2009 and 2019, 377 individuals of *C.guichenoti* were collected from the upper reaches of the Yangtze river basin. Tissue specimens of *C.guichenoti* were collected in strict accordance with relevant national laws and animal ethics requirements. During the collection process, a small amount of fish fin tissues was cut off and disinfected, and the fish were released into the river. Sampling sites cover most of the distribution range of the species. To ensure comparability, sample collection was considered using the same or similar as possible sampling points in the same water system or section. A total of five populations were collected in the Yanbian section of the Yalong River (YB), the Xufu section of the Jinsha River (SF), the Jiangan section of the Yangtze River (JA), the Luxian section of the Yangtze River (LX) and the Beibei section of the Jialing River (BB) in 2009. A total of seven populations were collected in the Xiqu section of Jinsha River (XQ), Hongmenchang section of Jinsha River (HMC), Longchi section of Minjiang River (LC), Caiba section of Yangtze River (CB), Xuzhou section of Yangtze River (XZ), Jiangyang section of Yangtze River (JY) and Liemian section of Jialing River (LM) in 2019 (Fig. 1).

To reduce the influence of different ages on the experimental error, 308 samples aged around 3 years were selected from 377 individuals, according to the relationship between age and body length (Ding 1994; Table 1). Total genomic DNA was extracted from the fin clips of *C.guichenoti* using PureLink Genomic DNA Mini Kit (Thermo Fisher Sentific, China).

Primers DL1 (ACCCCTGLCTCCCAAALC, Ta: 62 °C) and DH2 (ATCTTALCATCTTCAGTG, Ta: 62 °C) (Liu et al. 2014a) were set for the amplification of mtDNA control regions in all populations. The PCR conditions, electrophoresis and sequencing were as described in Liu et al. (2014b).

### Genetic diversity and population structure analysis

Haplotype number (H), Haplotype diversity (h), and nucleotide diversity (π) were used to estimate genetic diversity in mtDNA control regions (Nei 1987). Genetic diversity indices were calculated using DnaSP 3.2 (Rozas et al. 2003). Based on the mtDNA control region, a phylogenetic analysis was performed by Bayesian inference (BI) using *Coreiusheterodon* (Genbank no. FJ376082.1) and *Abbottinarivularis* (GenBank no. AP011257.1) as outgroups. The median connection network (MJN) method (Bandelt et al. 1999) was used by Network v. 3.1 to describe the relationships among all haplotypes. Pair genetic differentiation (FST) among populations were estimated using Genepop v. 2.4 (Zaigham et al. 2012).

### Population demography and landscape analysis

Population demography history was tested with mtDNA control region. First, Tajima’s D (Tajima 1989) and Fu’s FS (Fu 1997) were calculated for each population, under the recent population expansion model, using Arlequin v. 2.0. In addition, population history was studied by using DnaSP software to compare the mismatch distribution of each geographic sample with that of stable and expanded populations. Bayesian horizon map (BSP) was implemented in BEAST v. 1.5.4 (Drummond et al. 2005) and visualized in Tracer v. 1.5 (Drummond and Rambaut 2007). The final analysis was performed on 50 million generations, sampled every 2,000 generations, and aged for 5 million generations. The time for possible bottleneck or population expansion (T, calculated in substitution) is calculated from the relation S = 2UT (Rogers and Harpending 1992), where S is the pattern of mismatch distribution and U is the estimated mutation rate of the sequence.

Using the isolation with migration (IM) model in IMA2 program (Hey 2010), the migration rate is calculated based on the mtDNA control region. Running IMa2 required two steps (M mode and L mode). Firstly, in M mode, the model parameter function is estimated based on 6 ×10^8^ MCMC steps, according to the aging cycle of 200,000 steps. Secondly, the model parameter function estimated by M mode is used to estimate the offset rate in L mode.

To test the correlation between genetic differentiation and geographical distance, we performed Mantel test with mtDNA data. The significance of the correlation (*r*) between log-transformed genetic distance and log-transformed geographic distance was determined by using 2, 000 permutations of Distance Isolation (IBD) Web Service v. 3.1.6 (Jensen et al. 2005).

## Results

### Genetic diversity

**Figure 2.**
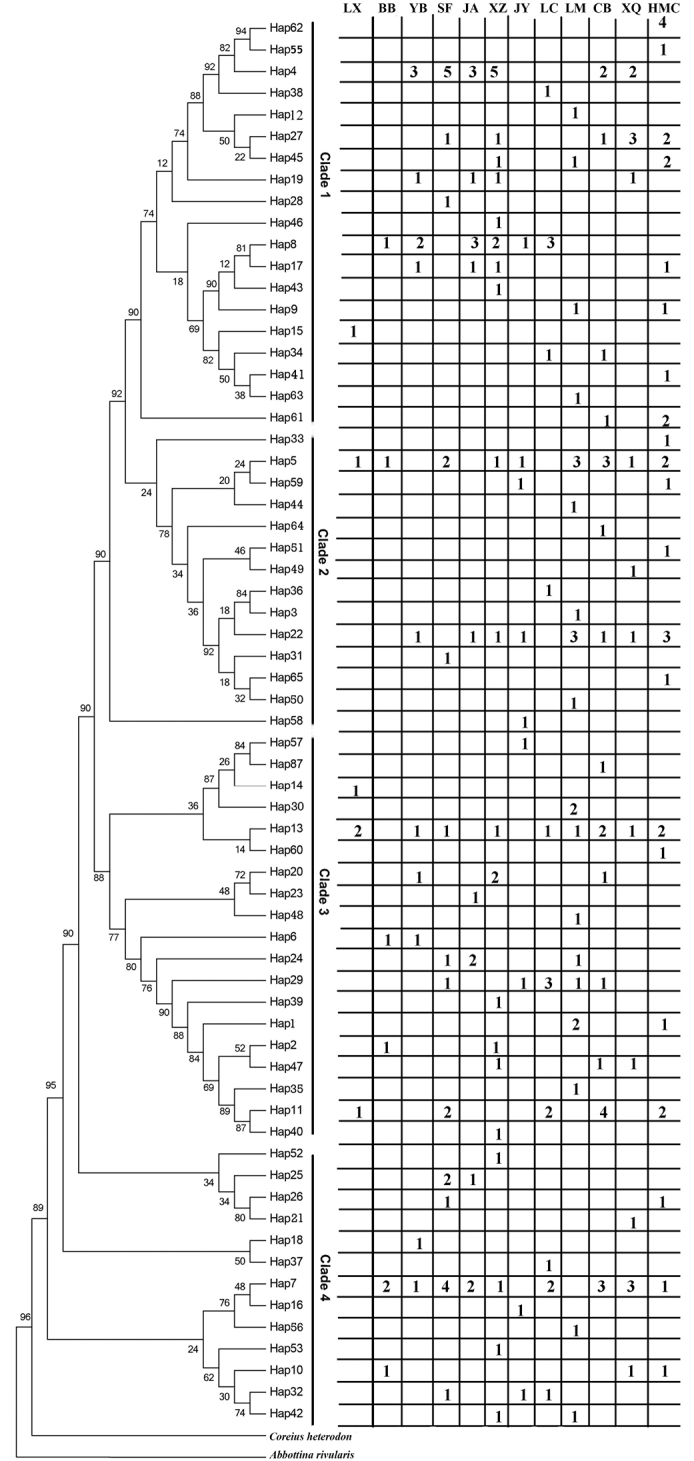
Phylogenetic trees of the mtDNA control region haplotypes in *C.guichenoti* reconstructed with Bayesian inference. Numbers at nodes represent Bayesian posterior probabilities and neighbor-joining tree. At the right side of the figure, the numbers represent the total of individuals from different sampling locations in each haplotype.

Genetic diversity analysis of 12 populations was performed based on mtDNA control region. Sequencing of 899 bp revealed 301 variable loci. The average base composition is A = 28.5%, T = 31.4%, C = 22.4% and G = 17.7%. A total of 65 haplotypes were identified from the control region sequences extracted from 308 individuals. Each population has some private haplotypes. However, with the exception of JY, Hap1 is shared in every population. LC, JA, XZ, HMC, and CB shared Hap3. YB, JA, SF, XZ, CB, and XQ share Hap4. All populations shared Hap5, except LC and YB. Each population shared Hap7, except for LX and JY. BB, JY, YB, JA, LC, and XZ shared Hap8 (Fig. 2). The haplotype diversity (h) varied from 0.869 to 0.901, and the nucleotide diversity (π) varied from 0.0031 to 0.0048. In both cases, the YB population had the highest number of individuals (Table 2). XZ and LM had the largest private number of haplotypes, 11 and 10, respectively. The numbers of private haplotypes were the least in BB (1) and JA (1). All differences of genetic variation (h and π) of 12 populations was not significant by paired *t*-test (*P* > 0.05).

**Table 2.** Population genetic diversity and structure statistics for the mitochondrial control region.

Population	H	h	π	Fis
LX	9	0.897	0.0038	0.146
BB	11	0.894	0.0042	0.254
YB	16	0.901	0.0047	0.114
SF	27	0.899	0.0038	0.112
JA	23	0.884	0.0044	0.227
XZ	28	0.874	0.0041	0.328
JY	13	0.891	0.0042	0.208
LC	25	0.878	0.0037	0.228
LM	30	0.876	0.0031	0.663*
CB	27	0.892	0.0038	0.377
XQ	19	0.869	0.0045	0.317
HMC	23	0.873	0.0044	0.757*

H: the number of haplotypes base on mtDNA; h: haplotype diversity based on mtDNA; π: nucleotide diversity based on mtDNA; Fis: mean inbreeding coefficients for 12 loci.

### Population Genetic structures

According to the sampling sites, the 12 populations were divided into three groups: upper group (YB, XQ and HMC), middle group (LX, JA, SF, JY, LC, CB, and XZ), and lower group (BB and LM). AMOVA analysis of five populations (JA, YB, BB, SF, and LX) collected in 2009 showed no significant molecular differences in mtDNA among populations, between populations and within populations (*P* > 0.05; Suppl. material 1: Table S1). AMOVA analysis of seven populations (JY, XZ, LM, CB, HMC, XQ, and LC) from 2019 showed that the intrapopulation variance (97.05%) was significant (*P* < 0.05, Suppl. material 1: Table S2).

The FST values of five populations (LX, BB, YB, SF, and JA) collected in 2009 were not significant (*P* > 0.05). The genetic differentiation between XQ and JA, HMC, and BB was not significant. Two neighborhood link trees were constructed based on the FST values in five populations (2009) and seven populations (2019). BB and LX populations clustered together, and SF, JA, and YB populations clustered in adjacency trees (Suppl. material 1: Fig. S1a). LC and JY, and LM and CB, are clustered together in adjacency trees, respectively (Suppl. material 1: Fig. S1b). HMC, XZ, and XQ populations are clustered in adjacent connection trees (Suppl. material 1: Fig. S1b).

**Figure 3.**
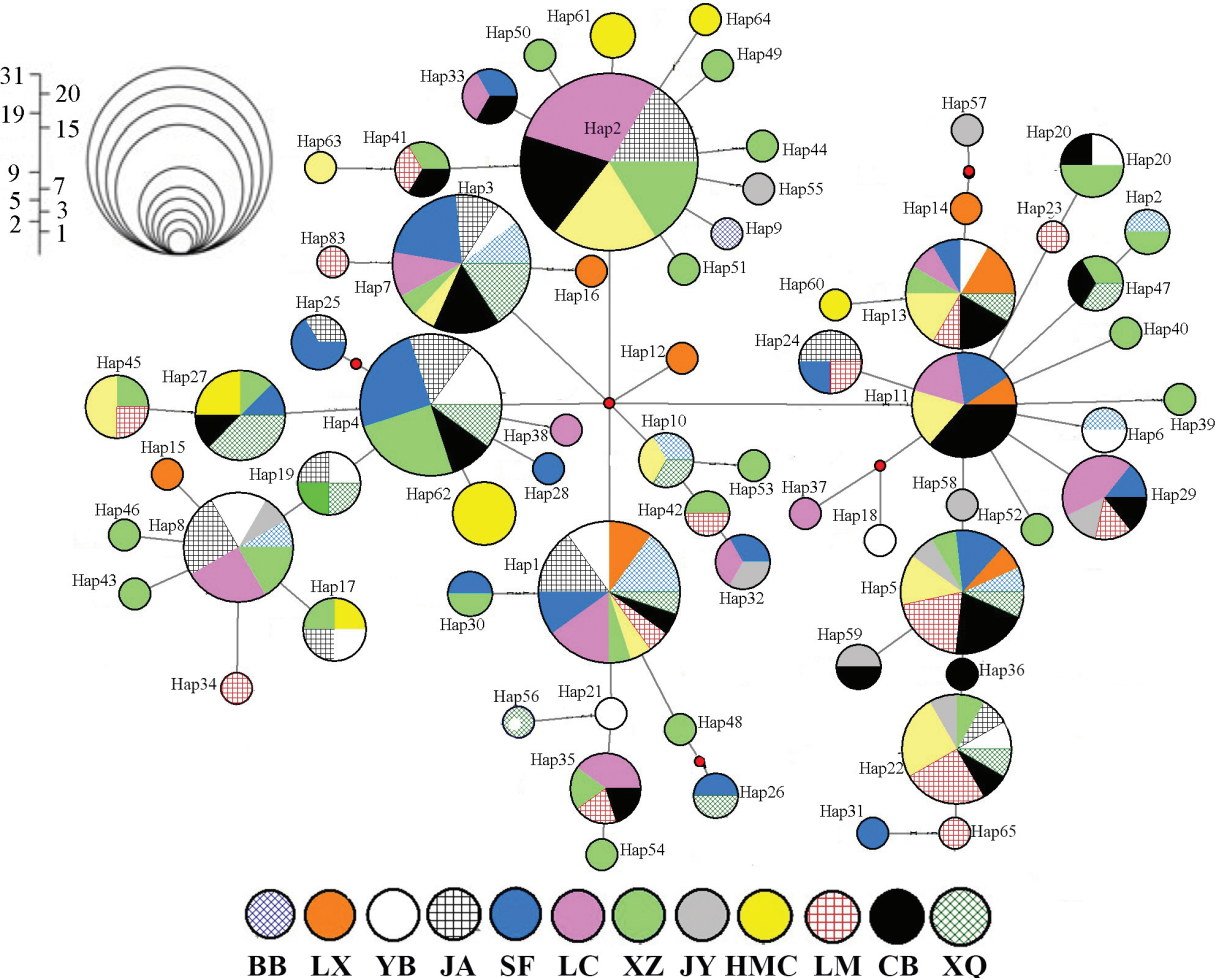
Median-joining network of the mtDNA control region haplotypes of *C.guichenoti*. The size of each circle indicates the relative frequency of the corresponding haplotype in the whole data set.

The results of mtDNA haplotype sequence analysis showed that there were four clades in the tree (Fig. 2), but no clear geographical distribution pattern was shown. Clades 1, 2, and 3, were composed of 12 population haplotypes. Clade 4 consists solely of 12 populations except for LX (Fig. 2). At the same time, the middle join network shows all single haplotypes from 12 populations that do not form a unique geographic structure of the haplotype network (Fig. 3). The four centers (Hap1, Hap3, Hap4, and Hap11), rich haplotypes, and other low-frequency-derived haplotypes were included in the haplotype network (Fig. 3). The frequency of Hap1 (*n* = 20), Hap3 (*n* = 32), Hap4 (*n* = 20), and Hap11 (*n* = 11) was different at different sites. Hap3 and Hap4 were not observed in the down group (BB and LM). The analysis of the aggregation method based on the IM model showed that the level of gene flow between populations was very limited (Suppl. material 1: Fig. S2). There was no significant gene flow in any of the five populations (Suppl. material 1: Fig. S2a; 2009) and seven populations (Suppl. material 1: Fig. S2B; 2019).

**Table 3.** Matrix of pairwise FST values calculated from the mtDNA control region data.

	LX	BB	YB	SF	JA	XZ	JY	LC	LM	CB	XQ
LX											
BB	0.026										
YB	0.036	0.013									
SF	0.030	0.042	0.011								
JA	0.023	0.064	0.032	0.043							
XZ	0.037	0.052	0.034	0.032	0.016						
JY	0.056	0.036	0.024	0.051	0.044	0.045					
LC	0.012	0.057	0.057	0.041	0.015	0.043	0.034				
LM	0.015	0.047	0.042	0.053	0.064	0.028	0.068	0.022			
CB	0.032	0.045	0.038	0.064	0.052	0.053	0.028	0.063	0.032		
XQ	0.061	0.026	0.018	0.074	0.064	0.065	0.046	0.059	0.118*	0.022	
HMC	0.023	0.067	0.054	0.024	0.017	0.014	0.028	0.113*	0.044	0.032	0.026

* FST values significantly different from zero (*P* < 0.05).

**Table 4.** Probabilities from tests (Wilconxon’s) for mutation drift equilibrium (bottlenecks) under three mutation models (IAM, TPM and SMM).

Population	Probability of Wilcoxon test		
	I.A.M	S.M.M	T.P.M
LX	0.1253	0.5441	0.3320
BB	0.0555	0.6762	0.7860
YB	0.0315*	0.7348	0.7327
SF	0.2237	0.2657	0.3388
JA	0.1436	0.9686	0.3026
XZ	0.0538	0.3246	0.1752
JY	0.1708	0.1028	0.1929
LC	0.0258*	0.0413*	0.0738
LM	0.0257*	0.0402*	0.0629
CB	0.1114	0.2026	0.1088
XQ	0.0452*	0.2324	0.0526
HMC	0.0384*	0.0438*	0.0635

*P < 0.05 (rejection of mutation drift equilibrium)

### Population demography and landscape genetic analysis

**Figure 4.**
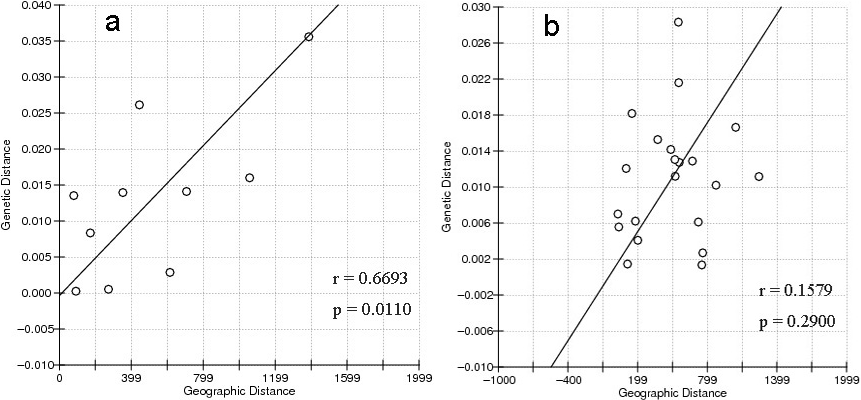
Isolation by distance (IBD) relationship among *C.guichenoti* wild populations in five populations collected in 2009 **a** and seven populations collected in 2019 **b**.

Mantel test showed that geographical distance was significantly associated with genetic differentiation in five populations (2009; Fig. 4a) and seven populations (2014–2019; Fig. 4b). However, in seven populations (2019; Fig. 4b), there was a weaker (than 2009) correlation between observed genetic differentiation and geographical distance (*r* = 0.1579, *P* = 0.2900).

The multichannel mismatch distribution, insignificant Tajima’s D and Fu’s FS values, and BSP analysis showed that SF, LX, BB, JA, YB, and XQ populations were relatively stable. However, LC and LM suffer genetic bottlenecks and XZ suffers dilatations. The bottleneck trend in the JY population and a weak population growth trend in the HMC and CB population.

## Discussion

### Changing of genetic diversity

In this study, we assessed genetic variation in twelve populations of *C.guichenoti* to understand the dynamics of the species’ genetic variability and its population structure and explore whether the rapid reduction of genetic variability will be observed in this species. Adaptive potential is related to genetic diversity, and the loss of genetic diversity can increase the possibility of population extinction. Previous work has highlighted the possible importance of genetic variability in determining the health of both individuals and, perhaps by implication, populations (Pritchard et al. 2000). Adults of *C.guichenoti* usually swim upstream when rivers are in flood, and after spawning they drift downstream (at lower elevations), suggesting that the upstream region may be the breeding and distribution center of the Yangtze River and its tributaries (Cheng et al. 2015). There was no significant difference in genetic diversity among the 12 populations by paired *t*-test (*P* > 0.05), indicating that there was no significant decrease in genetic diversity from 2009 to 2019, through the mean value of genetic diversity in 2009 was higher than that in 2019. Based on this result, it appears that the breeding of *C.guichenoti* has not been completely disrupted by dams. Some individuals may not need to go upstream to spawn and may be able to breed in scattered habitats, even if they are severely affected by dams.

### Population genetic structure and historical dynamics

The results of AMOVA analysis showed that the genetic differentiation between populations and between populations was limited (*P* > 0.05), and most of the total genetic variation occurred among individuals within groups, which was insignificant neither (*P* > 0.05). The FST values showed that the population structure of each sampling site was not significant. Moreover, these five groups do not form independent groups in terms of population structure, and each individual actually has an equal probability of belonging to any of these groups in any analysis. There was no obvious systematic geographic structure among the haplotypes of the samples from different geographical locations. The sequence differences within and between groups were small, indicating high genetic similarity among different groups and relatively shallow population history. The genetic differentiation of the five populations was significantly correlated with geographical distance. After spawning, this species usually flows downstream with floods, and when rivers are in flood, juvenile and adult fish subsequently swim upstream (Chen et al. 2002). These life history characteristics can facilitate gene communication between populations. Insignificant genetic differentiation among groups was also observed in *Leptobotiaelongata* (Bleeker, 1870) in the Yangtze River, and this was also attributed to spawning of drifting eggs and larvae (Liu et al. 2020). This is consistent with the lack of genetic population structure due to the spawning and life history traits of *C.guichenoti* and also might be related to the uncertainty of the distance that adults swim upstream.

However, different population structures were found in seven populations collected from 2019. There were significant differences in FST values between some populations collected from 2019. The seven populations did not form an independent cluster in population structure, but the composition of each population was different. Our AMOVA analysis showed that although the genetic differentiation between populations was very limited, there was significant genetic variation among individuals within populations (*P* < 0.05). Significant differences among all populations have not yet developed, but differences among individuals within populations are emerging. To explain this, the dam restricted the genetic links between the upstream and downstream populations of *C.guichenoti*, splitting them into small, isolated populations. A strong, though occasionally permeable, barrier to transmission may exist to limit the flow of genes between sites. In this case, the species’ habitat becomes fragmented, which not only affects the spawning and development environment of *C.guichenoti*, but also impedes upstream and downstream migration and gene exchange. Thus, weak correlation between the observed genetic differentiations and geographical distances was found in seven populations (2019, *P* > 0.05) while it is significant negative correlation in five populations (2009, *P* < 0.05). As the hydrological environment changes, the individuals within the population become different. Then there may be a strong demographic structure in the future. Furthermore, compared with large populations, small populations are more prone to inbreeding and loss of genetic diversity due to genetic drift and bottlenecks (Hoffman et al. 2009). In addition, the formation of reservoirs in the upper reaches of the Yangtze River has greatly changed the hydrological environment, which may damage the habitat and spawning grounds of *C.guichenoti*. Due to the lack of running water, larvae may not develop successfully. As a result, the abundance and genetic diversity of wild populations will inevitably decline.

### Suggestions for conservation

Though the decrease of genetic diversity was not significant from 2009 to 2019, the population structure of *C.guichenoti* showed a trend of change. If conservation and restoration measures will be not carried out, a loss of genetic diversity may occur in the future. Loss of genetic diversity will impair the ability of the population to respond to environmental changes, so conservation of genetic variation should be a priority in the recovery efforts of *C.guichenoti*, especially in light of recent habitat fragmentation due to dams.

Migration (increased gene exchange) can lead to gene rescue or recovery (Richards 2000). Increasing the population of a threatened population by culturing individuals may reduce the abundance of a wild population, but when properly applied, it can increase the genetic exchange of the species, which is a viable strategy for the conservation of the species. Various fish passage facilities have been developed to help species to cross these obstacles (Gow et al. 2011). Fishways are hydraulic structures that help fish to climb over obstacles such as dams and locks to spawn and migrate. Fishway plays an important role in fish conservation, but the corresponding design work is complex, which needs to consider the practical design criteria of hydraulic engineering, the path selection of fish swimming in fishway and the corresponding mortality evaluation of fish (Liu et al. 2012). Hydropower stations and researchers in China should devote great efforts in solving these problems, aiming for more precise quantitative predictions of efficient fish-passage and an optimum design of the hydraulic structure.

Furthermore, in order to maintain heterozygosity and genetic diversity, mature adults should be collected for artificial propagation from different locations across the distribution, especially considering the high genetic diversity among groups (An et al. 2016). Aquaculture conditions of hatcheries should be improved to increase the number of qualified parental fish and attention paid to the individual genetic relationship during artificial propagating. Restocking efforts also should strive to maintain genetic connectivity and exchange among local parental fish in the hatcheries to avoid inbreeding and increase genetic variation. Similarly, cultured juveniles and young fishes should be released into various habitats across the distribution as well (Clay 1995). Then, the population dynamics and genetic diversity of these wild populations should be monitored frequently, and protection measures be adjusted subsequently when needed.

## Supplementary materials

Supplementary material 1 Tables S1, S2, Figures S1, S2 Data type MS Word file Explanation note **Table S1.** Results of hierarchical AMOVA for five populations sampled in 2009 based on mitochondrial control region. **Table S2.** Results of hierarchical AMOVA for seven populations sampled in 2019 based on mitochondrial control region. **Figure S1.** Neighbor-joining tree based on FST. **Figure S2.** History in IM analyses for C.guichenoti. The boxes represent sampled and ancestral populations, horizontal lines represent splitting times and curved arrows represent migration. https://binary.pensoft.net/file/576666This dataset is made available under the Open Database License (http://opendatacommons.org/licenses/odbl/1.0/). The Open Database License (ODbL) is a license agreement intended to allow users to freely share, modify, and use this Dataset while maintaining this same freedom for others, provided that the original source and author(s) are credited.Dongqi Liu, Feng Lan, Sicai Xie, Yi Diao, Yi Zheng, Junhui Gong

## References

[B1] An R Li J Liang R Tuo Y (2016) Three-dimensional simulation and experimental study for optimizing a vertical slot fishway. Journal of Hydro-environment Research 12: 119–129. 10.1016/j.jher.2016.05.005

[B2] Bandelt HJ Forster P Rohl A (1999) Median-joining networks for inferring intraspecific phylogenies. Molecular Biology and Evolution 16: 37–48. 10.1093/oxfordjournals.molbev.a026036 10331250

[B3] Chen D Duan X Liu S Shi W Wang B (2002) On the dynamics of fishery resources of the Yangtze River and its management. Acta Hydrobiologia Sinica 26: 685–690. [In Chinese]

[B4] Cheng F Li W Klopfer M Murphy B Xie S (2015) Population genetic structure and its implication for conservation of *Coreiusguichenoti* in the upper Yangtze River. Environmental Biology of Fishes 98: 1999–2007. 10.1007/s10641-015-0419-z

[B5] Ding R (1994) The Fishes of Sichuan, China. Sichuan Publishing House of Science and Technology, Chengdu, China. [in Chinese]

[B6] Drummond AJ Rambaut A (2007) Beast: Bayesian evolutionary analysis by sampling trees. BMC Evolutionary Biology 7: e214. 10.1186/1471-2148-7-214 PMC2247476 17996036

[B7] Drummond AJ Rambaut A Shapiro B Pybus OG (2005) Bayesian coalescent inference of past population dynamics from molecular sequences. Molecular Biology and Evolution 22: 1185–1192. 10.1093/molbev/msi103 15703244

[B8] Fu Y (1997) Statistical tests of neutrality of mutations against population growth, hitchhiking and background selection. Genetics 147: 915–925. 10.1093/genetics/147.2.915 9335623 PMC1208208

[B9] Jiang ZG Jiang JP Wang YZ (2016) Red list of China’s Vertebrates. Biodiversity Science 24: 500–551. [In Chinese] 10.17520/biods.2016097

[B10] Hedrick PW Dowling TE Minckley WL Tibbets CA Demarais BD Marsh PC (2000) Establishing a captivebroodstock for the endangered bonytail chub (*Gilaelegans*). Journal of Heredity 91: 35–39. 10.1093/jhered/91.1.35 10739122

[B11] Hey J (2010) Isolation with migration models for more than two populations. Molecular Biology and Evolution 27: 905–920. 10.1093/molbev/msp296 19955477 PMC2877539

[B12] Hoffman JI Dasmahapatra KK Amos W Phillips CD Gelatt TS Bickham JW (2009) Contrasting patterns of genetic diversity at three different genetic markers in a marine mammal metapopulation. Molecular Ecology 18: 2961–2978. 10.1111/j.1365-294X.2009.04246.x 19500256

[B13] Jensen JL Bohonak AJ Kelley ST (2005) Isolation by distance, web service. BMC Genetics 6: 1–6. 10.1186/1471-2156-6-13 15760479 PMC1079815

[B14] Liu D Deng Y Li Z Li J Song Z (2014a) Isolation and characterization of 14 tetranucleotide microsatellite DNA markers in the elongate loach *Leptobotiaelongata* (Cypriniformes: Cobitidae) using 454 sequencing technology. Conservation Genetics Resources 6: 119–121. 10.1007/s12686-013-0021-7

[B15] Liu D Hou F Liu Q Zhang X Yan T Song Z (2015) Strong population structure of *Schizopygopsischengi* and the origin of *S.chengibaoxingensis* revealed by mtDNA and microsatellite markers. Genetica 143: 73–84. 10.1007/s10709-015-9815-8 25572029

[B16] Liu D LI X Song Z (2020) No decline of genetic diversity in elongate loach (*Leptobotiaelongata*) with a tendency to form population structure in the upper Yangtze River. Global Ecology and Conservation 23: e01072. 10.1016/j.gecco.2020.e01072

[B17] Liu D Wu J Deng L Gan W Du L Song Z (2014b) Development of microsatellite markers for *Leptobotiaelongata* (Cypriniformes: Cobitidae) using 454 sequencing and cross-species amplification. Pakistan Journal of Zoology 46: 1147–1151. 10.1007/s12686-013-0021-7

[B18] Liu D Zhou Y Yang K Zhang X Chen Y Li C Li H Song Z (2018) Low genetic diversity in broodstocks of endangered Chinese sucker, *Myxocyprinusasiaticus*: implications for artificial propagation and conservation. ZooKeys 792: 117–132. 10.3897/zookeys.792.23785 PMC6207638 30386163

[B19] Liu G Zhou J Zhou D (2012) Mitochondrial DNA reveals low population differentiation in elongate loach, *Leptobotiaelongata* (Bleeker): implications for conservation. Environmental Biology of Fishes 93: 393–402. 10.1007/s10641-011-9927-7

[B20] Liu J (2004) Quantitative Analysis of Yangtze River specific fish threatened and order of priority. China Environmental Science 24: 395–399. [In Chinese]

[B21] Nei M (1987) Molecular Evolutionary Genetics. Columbia University Press, New York, USA. 10.7312/nei-92038

[B22] Pritchard JK Stephens M Donnelly P (2000) Inference of population structure using multilocus genotype data. Genetics 155: 945–959. 10.1093/genetics/155.2.945 10835412 PMC1461096

[B23] Richards CM (2000) Inbreeding depression and genetic rescue in a plant metapopulation. American Naturalist 155: 383–394. 10.1086/303324 10718733

[B24] Rogers AR Harpending H (1992) Population growth makes waves in the distribution of pairwise genetic differences. Molecular Biology and Evolution 9: 552–569. 10.1093/oxfordjournals.molbev.a040727 1316531

[B25] Rozas J Sanchez DJC Messeguer X Rozas R (2003) DnaSP, DNA polymorphism analyses by the coalescent and other methods. Bioinformatics 19: 2496–2497. 10.1079/9780851994758.0139 14668244

[B26] Tajima F (1989) Statistical method for testing the neutral mutation hypothesis by DNA polymorphism. Genetics 123: 585–595. 10.1093/genetics/123.3.585 2513255 PMC1203831

[B27] Zaigham A Basharat A Anjum NS (2012) Antimicrobial activity of biocides against different microorganisms isolated from biodeteriorated paints. Pakistan Journal of Zoology 44: 576–579. 10.1670/11-013

[B28] Zhang F Tan D (2010) Genetic diversity in population of largemouth bronze gudgeon (*Coreiusguichenoti* Sauvage et Dabry) from Yangtze River determined by microsatellite DNA analysis. Genes & Genetic Systems 85: 351–357. 10.1266/ggs.85.351 21317547

