# Supplementary material for: Dynamic genetic diversity and population structure of Coreiusguichenoti
Source: Zookeys. 2021 Aug 11;1055:135–48. doi: 10.3897/zookeys.1055.70117 (PMC8376836; doi:10.3897/zookeys.1055.70117)

**Table S1.** Results of hierarchical AMOVA for five populations sampled in 2009 based on mitochondrial control region.

| Source of variation | DF | SS | VC | %V | *P* |
| --- | --- | --- | --- | --- | --- |
| Among groups | 3 | 1.976 | 0.073 | 4 | 0.898 |
| Among populations | 4 | 4.142 | 0.033 | 0.7 | 0.852 |
| Within populations | 108 | 162.45 | 1.5423 | 95.3 | 0.763 |

Abbreviations: DF, degrees of freedom; SS, Sum of squares; VC, variance component; %V, percent of variance.

**Table S2.** Results of hierarchical AMOVA for seven populations sampled in 2019 based on mitochondrial control region.

| Source of variation | DF | SS | VC | %V | *P* |
| --- | --- | --- | --- | --- | --- |
| Among groups | 3 | 6.848 | 0.0789 | 1.43 | 0.206 |
| Among populations | 5 | 7.464 | 0.0314 | 1.54 | 0.452 |
| Within populations | 224 | 433.37 | 1.9439 | 97.03 | 0.042 |

Abbreviations: DF, degrees of freedom; SS, Sum of squares; VC, variance component; %V, percent of variance.

**Figure S1.** Neighbor-joining tree based on FST values.
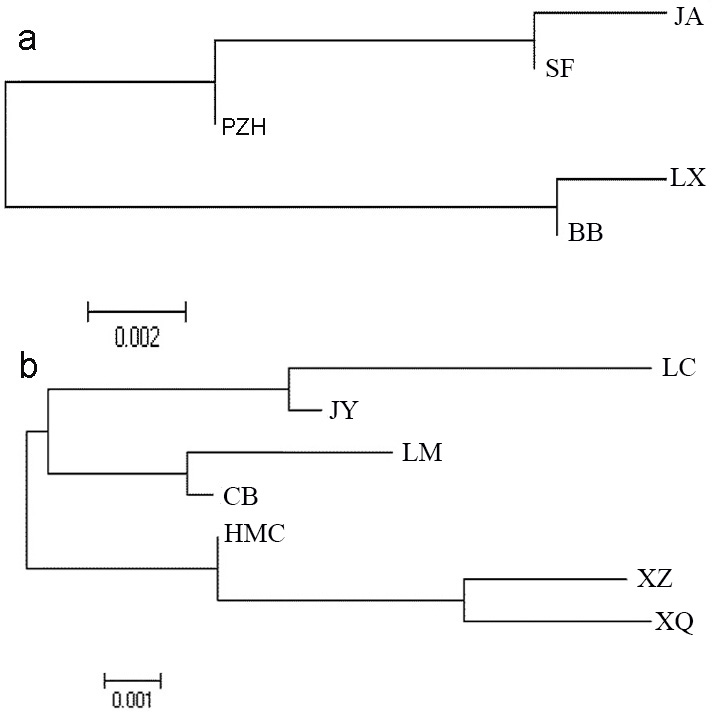
**.**

Figure S2. History in IM analyses for *C. guichenoti*. The boxes represent sampled and ancestral populations, horizontal lines represent splitting times and curved arrows represent migration. Time is represented as depth on the vertical axis, with the sampled population names at the top of the figure at the most recent time point. For the figure, the 95% highest posterior density (HPD) intervals are shown with arrows in gray for population sizes (box widths) and splitting times (dotted lines).


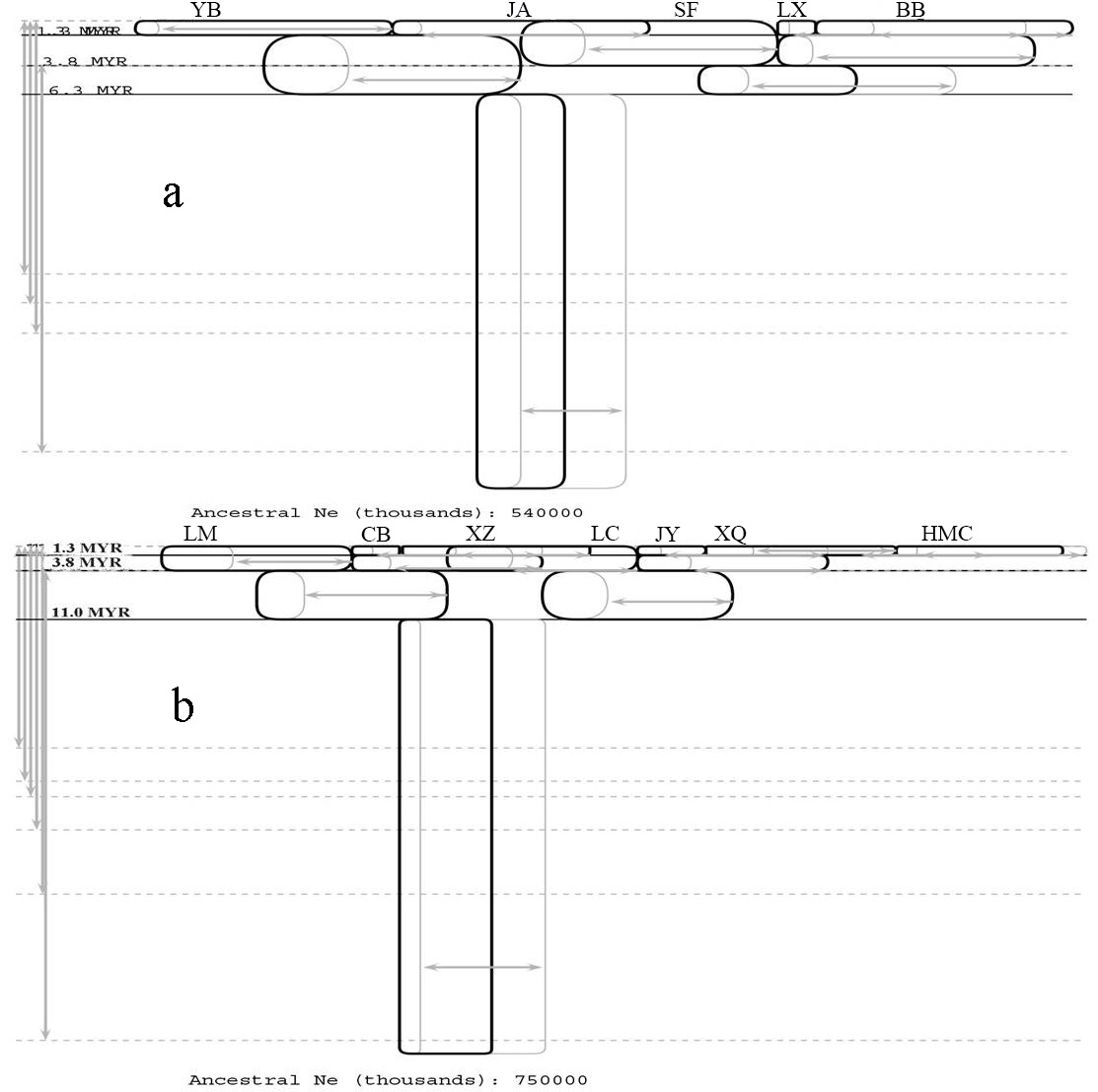

Supplement: Supplementary material 1 — Tables S1, S2, Figures S1, S2 [file zookeys-1055-135-s001.doc]
